# An attempt to optimize the outcome of penetrating keratoplasty in congenital aniridia-associated keratopathy (AAK)

**DOI:** 10.1007/s10792-021-01982-z

**Published:** 2021-07-29

**Authors:** C. J. Farah, F. N. Fries, L. Latta, B. Käsmann-Kellner, B. Seitz

**Affiliations:** 1 Department of Ophthalmology, Saarland University Medical Center, 100 Kirrbergerstr., Building 22, 66421 Homburg, Saar Germany; 2 Dr. Rolf M. Schwiete Center for Limbal Stem Cell Research and Congenital Aniridia, Saarland University Medical Center, Homburg, Saar Germany

**Keywords:** Aniridia-associated keratopathy, Penetrating keratoplasty, Limbal stem cell deficiency, Amnion membrane transplantation, Autologous serum

## Abstract

**Purpose:**

To propose an optimized microsurgical and medical approach to reduce the risk of complications after penetrating keratoplasty (PKP) in patients with aniridia-associated keratopathy (AAK).

**Methods:**

Retrospective observational case series of 25 PKP performed in 16 patients with AAK. Preoperative indications were endothelial decompensation and vascularized scars (68%) or graft failure (32%) due to limbal stem cell deficiency. The optimized approach included a combination of a small corneal graft size (around 7.0 mm), interrupted 10–0nylon sutures, simultaneous AMT as a patch, large bandage contact lens, temporary lateral tarsorrhaphy, postoperative autologous serum eye drops, and systemic immunosuppression. Main outcome measures included: visual acuity, transplant survival, and complications encountered during follow-up of 107 weeks on average.

**Results:**

A complete modified keratoplasty scheme was used in 10 of 25 PKP (group 1), while at least one of the modifications was missing in the other 15 PKP (group 2). After 8 weeks of follow-up, the epithelium was closed in 23 eyes. Visual acuity improved in 19 eyes at 6 months of follow-up, and remained stable in six eyes. None of the eyes showed a decrease in visual acuity. At the last post-operative follow-up, this visual improvement persisted in 14 eyes and graft survival rate after 156 weeks (3 years) was 69% in group 1 versus 44% in group 2 (*p* = 0.39, log-rank test). Secondary corneal neovascularization (8%), scarring (4%), ulcer (4%), or graft rejection (8%) happened mostly in the second group which was missing at least one of the suggested modifications.

**Conclusions:**

PKP in congenital aniridia must be considered as a high-risk keratoplasty. An optimized therapeutic approach seems to be promising in order to reduce the postoperative complication rate in these most difficult eyes.

## Introduction

First described as “congenital irideremia” in the nineteenth century, congenital aniridia is a rare (1:60.000–1:90.000) pan-ocular disease that can be differentiated in two major categories depending on the presence of a PAX-6-Gene mutation [1–4]. Among those with a mutation of the PAX-6-Gene, frequent mutations involve point mutations and deletions. A deletion of the short arm of chromosome 11(p13) may be autosomal dominant or sporadic, and to a lesser extent autosomal recessive, e.g., in the Gillespie syndrome [5]. A PAX-6-Gene mutation is more frequently associated with ocular complications compared to aniridia triggered by other mutations than the PAX-6-Gene [2, 3]. Congenital aniridia is linked to different malformations such as iris, macular or optic nerve hypoplasia, but also to deteriorating progressive major ocular dysfunctions such as limbal stem cell deficiency, premature onset of cataract, and secondary glaucoma that can lead to blindness throughout life [2, 3, 6–8]. The incidence and severity of aniridia-associated keratopathy (AAK) increases with age affecting about 20–30% of those patients and leads to corneal opacities, scarring, and vascularization due to a unique form of limbal stem cell deficiency [2, 9–11].

With time, it may result in corneal ulcers, dense vascularized scars, or endothelial decompensation (especially after complicated cataract and/or glaucoma surgery), where a penetrating keratoplasty becomes indicated [8]. Persisting epithelial defects, suture loosening, and an increased risk of graft rejection are typical postoperative complications in those high-risk keratoplasties [8]. We hypothesized that the combination of a small corneal graft size, interrupted sutures, simultaneous amniotic membrane transplantation (AMT) as a patch [12, 13], large bandage contact lens, temporary lateral tarsorrhaphy, postoperative autologous serum eye drops [14], and systemic immunosuppression [15, 16] may improve the outcome after PKP in congenital aniridia.

## Patients and methods

This study is a retrospective observational case series of 25 penetrating keratoplasties (PKP) performed in 20 eyes of 16 patients with AAK at the Department of Ophthalmology of the Saarland University Medical Center in Germany between 2012 and 2019. Four patients received a bilateral surgery. All procedures were performed in accordance with the ethical standards of the 1964 Helsinki Declaration and its later amendments. Transplantation had been proposed as a last resort therapy in corneas with severe endothelial decompensation and stromal scars (68%) and graft failure (32%), due to limbal stem cell deficiency with no success of conservative treatments (stage IV or V according to Yazdanpanah et al. [17]).

**Fig. 1:**
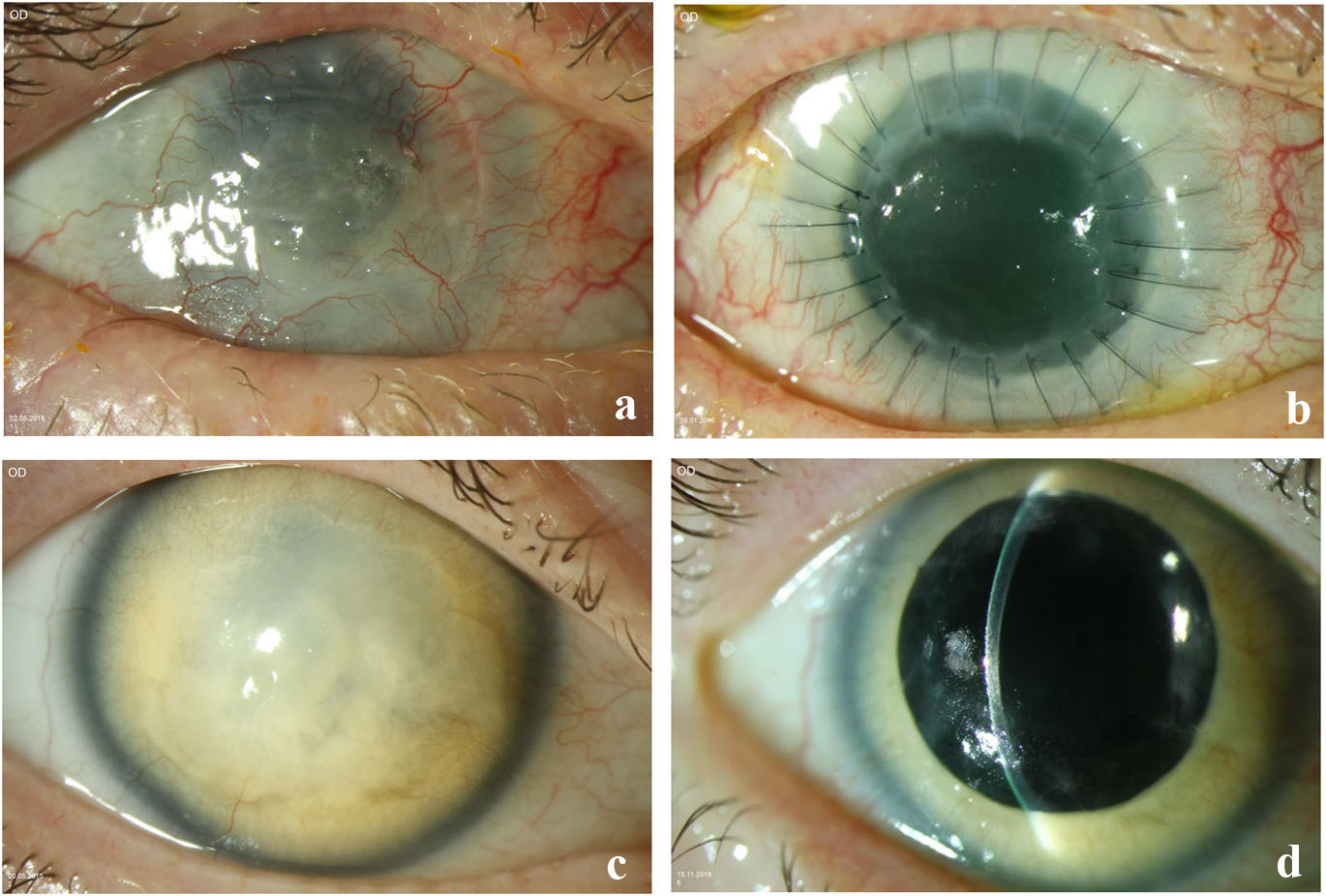
**a** Patient 1: Preoperative picture with decompensated and severely vascularized graft, 6 years after penetrating keratoplasty. Visual acuity: Hand motion (logMar 2.7). **b** Patient 1: 8 months postoperative picture with 26 interrupted sutures after repeat penetrating keratoplasty with Barron trephine (7.00/7.25 mm). Visual acuity has reached logMar 2.3. **c** Patient 2: 64-year old woman with corneal decompensation, vascularized cornea, secondary amyloidosis, and premature cataract in congenital aniridia. Visual acuity: Hand motion (logMAR 2.7). **d** Patient 2: Clear corneal graft, 18 months after classical excimer laser-assisted triple procedure (7.0/7.1 mm) and suture removal. Visual acuity improved to logMAR 1.0

The mean age during keratoplasty was 52 ± 8 (from 26 to 64) years, and most eyes (92%) had a history of previous surgeries (Table 1). We defined a small graft as ranging from 6.0 to 7.5 mm diameter. Two types of 10–0 nylon sutures were used as follows: double-running cross-stitch sutures according to Hoffmann versus 24–32 interrupted sutures (Fig. 1). Amniotic membranes were collected from healthy women with their consent and properly processed in the eye bank before transplantation [18]. They were transplanted at the end of the keratoplasty as a single 16-mm layer membrane with the stromal side facing the corneal graft and fixed with a running 10–0 nylon episcleral suture as a patch [12]. The membrane was covered with a large 17-mm bandage contact lens, and both sutures and contact lens were removed after a period of 4–6 weeks. Temporary lateral tarsorrhaphy was performed using 5–0 silk and left typically for 4–6 weeks after PKP.

**Table 1:** Characteristics of 16 patients with congenital aniridia (25 keratoplasties)

Patients	*n* = 16 (100%)
Males	6 (37,5%)
Females	10 (62,5%)
Genetics	*n* = 16 (100%)
PAX-6	7 (43,75%)
WAGR(O)	1 (6,25%)
Genetically not analyzed	8 (50%)
Type of Keratoplasty	*n* = 25 (100%)
First keratoplasty (with or without simultaneous pannus removal)	13 (52%)
Repeat keratoplasty (with or without simultaneous pannus removal)	5 (20%)
Classical triple procedure (combined with cataract surgery and intraocular lens implantation)	4 (16%)
Pole-to-pole surgery (keratoplasty combined with lens and vitreoretinal surgery)	2 (8%)
HLA-typed keratoplasty	1 (4%)
History of previous surgeries before keratoplasty (Multiple choices possible)	*n* = 25 (100%)
Glaucoma surgery	11 (44%)
Trabeculotomy	3 (12%)
Cyclophotocoagulation	9 (36%)
Ahmed valve	5 (20%)
Corneal surgery	12 (48%)
Pannus removal (“pannectomy”)	3 (12%)
Phototherapeutic keratectomy (PTK)	1 (4%)
Penetrating keratoplasty (in domo)	5 (20%)
Penetrating keratoplasty (ex domo)	3 (12%)
Cataract surgery	8 (32%)
Retinal surgery	4 (24%)
None	2 (8%)

After exclusion of systemic infectious diseases, 100%-concentrated autologous serum eye drops were prepared [19, 20]. During the first postoperative days, they were applied hourly alternating with preservative-free hyaluronic acid containing artificial tear eye drops. A topical antibiotic coverage for at least 4 weeks with ofloxacin or moxifloxacin eyedrops five times daily was necessary to prevent infections until complete epithelial healing was achieved, and the bandage contact lens was removed, typically after 4 weeks. An additional long-term therapy with topical acyclovir (five times daily) and systemic acyclovir (starting with 400 mg five times daily for 6 weeks, then two times daily for 1 year) was given to patients with history of herpetic keratitis.

Postoperative topical corticosteroids (starting at five times daily, being reduced by 1 drop every 6–8 weeks) and systemic corticosteroid (prednisolone, prednisolonacetat, and methylprednisolone) were slowly tapered over 4 weeks, starting at 100 mg daily and being reduced by 20 mg every second day until 20 mg daily, then being reduced slowly. A systematic preoperative evaluation for long-term use of immunosuppression with mycophenolate mofetil or cyclosporin A and systemic follow-up were established in collaboration with the family physician, and doses were adapted to the general condition of the patient.

Transplantations were combined with simultaneous cataract or vitreoretinal surgeries where indicated. Characteristics of recipients are shown in Table 1. The optimized approach included a combination of a small corneal graft size (around 7.0 mm), interrupted 10–0 nylon sutures, simultaneous AMT as a patch, large bandage contact lens, temporary lateral tarsorrhaphy, postoperative autologous serum eye drops, and systemic immunosuppression. Main outcome measures included: visual acuity, transplant survival, and complications encountered during the follow-up of 107 weeks on average.

For statistical analysis, eyes were separated into two groups based on the therapeutic scheme. A complete, modified keratoplasty scheme was used in 10 of 25 PKP (group 1). The second group included all other PKP, where at least one modification of the modified keratoplasty scheme was missing (group 2). Techniques used are illustrated in Table 2.

**Table 2:** Techniques used in 25 keratoplasties (Multiple choices possible)

Diameter of recipient openings (diameter in mm)	*n* = 25 (100%)
6.5 mm	1 (4%)
7.0 mm	14 (56%)
7.5 mm	9 (36%)
12.0 mm (Limbo-keratoplasty)	1 (4%)
Sutures	*n* = 25 (100%)
Interrupted sutures	18 (72%)
Double-running cross-stitch sutures according to Hoffmann (1976)	7 (28%)
Use of simultaneous amnion membrane transplantation (Patch)	*n* = 25 (100%)
Yes	24 (96%)
No	1 (4%)
Use of simultaneous lateral tarsorrhaphy	*n* = 25 (100%)
Yes	14 (56%)
No	11 (44%)
Use of postoperative autologous serum eye drops primarily	*n* = 25 (100%)
Yes	19 (76%)
No	6 (24%)
Use of long-term immunosuppressive therapy	*n* = 25 (100%)
Mycophenolate mofetil	20 (80%)
Cyclosporin-A	3 (12%)
None	2 (8%)

Statistical analysis was performed using SPSS v. 20.0.0 (IBM Corp., Armonk, NY, USA). Visual acuity was recorded in decimal and converted into logMAR before analysis. Graft survival was analyzed using the Kaplan–Meier method and log-rank test, which is appropriate to compare small sample sizes and unequal censoring groups.

## Results

### Visual acuity

At 6 months of follow-up, the mean visual acuity (VA) improved from logMAR 2.18 to logMAR 1.65. None of the eyes showed a decreased VA. On the other hand, the VA remained unchanged in six eyes and improved in 19 eyes. The last examination was made on average 107 weeks postoperatively with a mean VA of logMAR 1.69 in group 1 and logMAR 1.77 in group 2.

All patients reported a subjective improvement in their visual acuity, either by increased clarity of the image, or by decreased visual discomfort caused by corneal optical phenomena such as glare. However, no specific questionnaires have been used to standardize subjective assessment of patients. This visual improvement persisted in 14 eyes until the end of follow-up (107 weeks on average).

### Graft survival

**Fig. 2:**
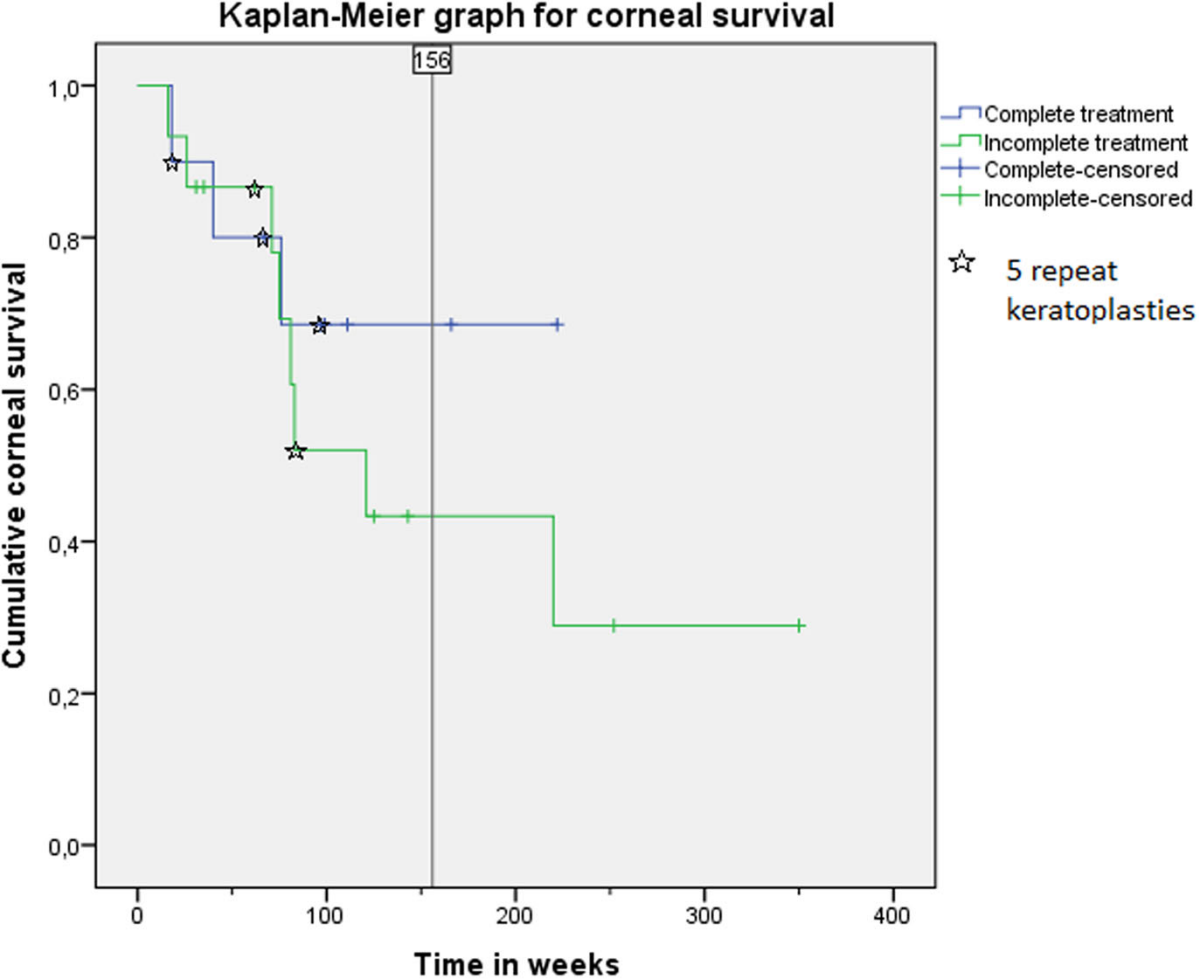
Survival of corneal grafts in group 1 (complete treatment) versus group 2 (incomplete treatment). Graft survival rate after 156 weeks (3 years) was 69% in group 1 and 44% in group 2, (*p* = 0.39, logrank test)

At 8 weeks of follow-up, the epithelium was closed in 23 eyes and only 2 eyes needed a second AMT. Graft survival was referred to as a clear and transparent graft, without endothelial decompensation scars or corneal opacities. Graft survival is demonstrated in a Kaplan–Meier chart (Fig. 2). The mean postoperative follow-up was 119 weeks in group 1 and 216 weeks in group 2. Graft survival rate after 156 weeks (3 years) was 69% in group 1 and 44% in group 2 (*p* = 0.39, log-rank test). The median graft survival time was 97 weeks in group 1 and 81 weeks in group 2.

### Adverse events

While only two eyes needed a repeat keratoplasty in group 1 and 3 eyes in group 2, severe corneal complications such as graft rejection (8%), anterior segment fibrosis syndrome (4%) or graft ulcer (16%), mostly occurred in group 2. Other, less severe corneal complications such as persistent epithelial defects (20%) and premature suture removal/replacement (28%) were present in both groups (Table 3).

**Table 3:** Corneal complications during follow-up. *n* = 25 keratoplasties (100%). Group 1: Complete therapeutic modified scheme (*n* = 10). Group 2: Incomplete therapeutic scheme (*n* = 15)

Persistent epithelial defect	*n* = 5 (20%)
Group 1	3 (12%)
Group 2	2 (8%)
Premature suture removal/replacement	*n* = 7 (28%)
Group 1	3 (12%)
Group 2	4 (16%)
Corneal endothelial decompensation	*n* = 4 (16%)
Group 1	2 (8%)
Group 2	2 (8%)
Immunological graft rejection	*n* = 2 (8%)
Group 1	0 (0%)
Group 2	2 (8%)
Graft ulcer/neovascularization/scarring/anterior segment fibrosis syndrome	*n* = 5 (20%)
Group 1	1 (4%)
Group 2	4 (16%)

A second AMT as patch was used to treat persistent epithelial defects in five eyes. Loose corneal sutures were removed as quickly as possible to prevent infiltrates and infections. Graft rejections were primarily treated with topical intracameral and systemic steroids, followed by a repeat keratoplasty, if needed. The two eyes that required a repeat keratoplasty after graft rejection had contraindications to systemic immunosuppressive therapy.

Extracorneal complications, regardless of the chosen therapeutic scheme, were changes in ocular pressure (hypo- and hypertension) (20%), retinal detachment (4%), retinal vein occlusion (4%), and intraocular lens luxation (4%). Those complications were handled according to the respective German guidelines.

## Discussion

The development of microsurgical techniques and the knowledge of limbal stem cell function has led to a considerable improvement in the treatment and visual prognosis of eyes with congenital aniridia over the years [3–9, 21–23]. Each small improvement is helpful for those progressively visually impaired patients. The global therapeutic approach should always consider a high risk of concomitant glaucomatous damage with irreversible optic atrophy due to a mispositioning of the ciliary body processes toward the iris stump and a very short ciliary body [2, 7, 24].

In well-selected cases, the treatment of AAK with PKP has shown to be beneficial, even though the post-operative complications turned out to be more frequent in those high-risk eyes [6, 8, 25]. Our modified treatment scheme appears to reduce the severe postoperative complications and improve visual prognosis as well as graft survival in the mid-term follow-up.

At first glance, the Kaplan–Meier curve may suggest a different trend between the groups, but the log-rank test could not detect a statistically significant difference between the two groups in terms of graft survival (*p* = 0.39). We attribute this to the low number of cases, which is due to the rarity of the disease. Based on our clinical experience, we refrain from randomizing patients as we do not want to deprive them of what we believe to be the optimal treatment nowadays.

We explain this difference by the following preventive measures: the use of interrupted sutures allows a quick removal of loose sutures at the slit lamp without risking graft slippage, and thus reduces the risk of infections and secondary immune reactions [27]. A temporary lateral tarsorrhaphy combined with simultaneous AMT as a patch and 17-mm bandage contact lens considerably reduces postoperative epithelial defects due to a mechanical protective effect [12, 28, 29].

Furthermore, the amnion membrane supports epithelialization, it has anti-fibrotic effects (due to a reduced expression of TGF β1, β2, β3 isoforms and TGF-beta receptor II), and anti-inflammatory effects (through inhibition of proinflammatory cytokines). Moreover, anti-angiogenic effects (due to production of thrombospondin-1, endostatin and tissue inhibitors of metalloproteases), and immunomodulatory effects have been reported [12, 13, 18, 26]. Similar positive effects have been described for the autologous serum eye drops [13, 18, 25]. Due to the higher risk of graft rejection, a systemic immunosuppressive therapy proved to be useful in most patients [15, 16].

The limitations of the study are the small sample size due to the rare condition and the lack of long-term observation periods to demonstrate a significant difference between groups. A prospective randomized study is not possible for ethical reasons. As our proposed therapeutic scheme does not directly address the cause of AAK, it could be combined with a prior transplantation of allogenic limbal stem cells, even though limbal stem cell transplantation alone in patient with AAK has been significantly associated with progression of limbal stem cell deficiency severity and visual impairment [30]. However, the survival curve looks promising, with a major separation after only 2 years of follow-up. We may expect an average graft survival of 50% at 5 years for the group which was treated with the complete scheme, and only 30% for the other group (Fig. 2). This highlights the difficulty of surgical management in AAK compared to other corneal pathologies with limbal stem cell deficiency such as herpes keratitis (survival rate of 90% after 2 years and 49% after 10 years), while chemical burn-associated limbal stem cell deficiency could best benefit from our modified scheme to enhance graft survival (survival rate of 35% after 5 years and 14% after 10 years) [31, 32]. Moreover, the four patients that received a bilateral surgery showed a better graft survival in the eyes who benefited from the optimized treatment scheme. This may also speak in favor of the efficacy of our modified scheme.

Although the Boston keratoprosthesis shows promising results in small series of patients with AAK, other complications such as retroprosthetic membrane formation or device extrusion exists [6, 33]. Regarding the limbo-keratoplasty, Lang et al. reported a median graft survival of 3.2 years over a long-term observation, indicating that this might also be a favour of approach in “partial” limbal stem cell deficiency [34].

In conclusion, an optimized high-risk PKP approach seems to be promising to reduce the postoperative complication rate in these most difficult eyes with congenital aniridia. Furthermore, an extension to treat other high-risk corneal pathologies with limbal stem cell deficiency seems promising (e.g., chemical burn).

## Data Availability

The data were attached to the manuscript as a supplementary file.
